# Adverse Childhood Experiences and Subjective Cognitive Decline Among Transgender Adults

**DOI:** 10.1111/jnu.13047

**Published:** 2025-01-29

**Authors:** Jobina Chiow, Ethan C. Cicero

**Affiliations:** ^1^ Nell Hodgson Woodruff School of Nursing, Emory University Atlanta Georgia USA

**Keywords:** adverse childhood experiences, cognitive decline, gender identity, gender minority, subjective cognitive decline, transgender

## Abstract

**Introduction:**

Adverse childhood experiences (ACEs) are associated with an increased risk of developing chronic health conditions, including Alzheimer's disease and related dementias (ADRD) and subjective cognitive decline (SCD), self‐reported confusion/memory loss, and an early clinical manifestation of ADRD. While ACEs and SCD have both been individually studied in transgender and nonbinary (TGN) adults, no study has examined the relationship between the two among this population. This study sought to establish the prevalence of ACEs and their association with SCD among TGN adults.

**Design:**

Cross‐sectional, secondary analysis of publicly available data.

**Methods:**

2019–2021 Behavioral Risk Factor Surveillance System data, representing 16 US states that assessed ACEs, SCD, and self‐reported gender identity were used to determine the association between ACEs and SCD among TGN adults aged 45+ (*N* = 206). Pearson's chi‐squared/Fisher's exact tests assessed the association between ACEs (individual, categorical, sum score) and SCD. Crude and adjusted odds ratios (aORs) along with 95% confidence intervals (CIs) were calculated to investigate the associations between ACEs and SCD.

**Results:**

18% (*n* = 38) of TGN adults in the sample endorsed SCD, 60% (*n* = 120) experienced any ACE, 20% (*n* = 41) 1 ACE, and 18% (*n* = 37) experienced > 4 ACEs. Nearly 50% experienced childhood abuse (*n* = 94) or household dysfunction (*n* = 92). Among those with SCD, 34% (*n* = 13) reported > 4 ACEs, and 73% (*n* = 26) reported childhood abuse or household dysfunction (*n* = 27). Most ACES were associated with and increased the risk of SCD, even after adjusting for BRFSS year, age, race, education, and employment. The odds of SCD increased 40% as the number of ACEs increased (aOR = 1.4, 95% CI: 1.2–1.6, *p* < 0.0001). The odds of SCD were higher with childhood abuse (aOR = 4.3, 95% CI: 1.88–10.02, *p* < 0.01) or household dysfunction (aOR = 4.7, 95% CI: 2.00–11.07, *p* < 0.01).

**Conclusion:**

ACEs increase the risk of SCD among TGN adults. Gender‐affirming and trauma‐informed nursing care are important, and screening and interventions for ACEs and SCD are needed to help reduce the risk of SCD and ADRD.

**Clinical Relevance:**

Examining how adverse childhood experiences impact different aspects of health, including brain health, is important to nursing practice as it can provide clinical care strategies and identify interventions to specifically address ways to improve the health and well‐being of transgender and nonbinary people.

## 1 Introduction

Dementia is an overarching term that describes a group of symptoms that characterize difficulties with memory, language, problem‐solving, and other thinking skills (Alzheimer's Association 2023). Alzheimer's disease, the most common cause of dementia, is a potentially fatal, progressive, and degenerative brain disease in which neurons in the brain are damaged, especially in areas responsible for memory, language, and thinking (Alzheimer's Association 2023). As time progresses, an individual's ability to function independently day‐to‐day slowly deteriorates, making activities that were once done with ease more difficult to do.

In 2023, 6.7 million Americans aged 65 or older were estimated to be living with Alzheimer's disease, and by 2060, the number of Americans living with Alzheimer's disease and related dementias (ADRD) is expected to be 13.8 million people (Alzheimer's Association 2023). Many risk factors contribute to the development of ADRD, including but not limited to older age, family history, cardiovascular disease, hypertension, obesity, diabetes, smoking, traumatic brain injury, education, and exposure to environmental pollutants, (Alzheimer's Association 2023). In addition to these risk factors, identifying as a sexual or gender minority (SGM) person or experiencing subjective cognitive decline (SCD) or adverse childhood experiences (ACEs) may increase one's risk of ADRD (Alzheimer's Association 2023; Flatt et al. 2021; Gould et al. 2012). SGM is an academic term used to describe individuals who identify as lesbian, gay, bisexual, queer, or another sexuality other than heterosexual (sexual minority) or transgender, nonbinary, or gender expansive (gender minority) (Cicero et al. 2020a, 2020b). The term transgender describes people whose gender identity is not aligned with the sex they were assigned at birth (Cicero et al. 2020a, 2020b). The transgender population includes individuals who identify within the gender binary (e.g., transgender men, transgender women, transmasculine, transfeminine) as well as those who identify outside of the gender binary (e.g., nonbinary, genderqueer) (National Center for Transgender Equality 2023). In this paper, we use the term “transgender” (TGN) as an inclusive term that represents binary and nonbinary identities used to refer to people who belong to the transgender population.

Research exploring ADRD among TGN adults is limited (Flatt et al. 2021, 2022); however, two studies provide insight into the prevalence of ADRD among this population. Researchers examining electronic health records and claims data from the OneFlorida Clinical Research Consortium found more TGN adults (3.5%) had a diagnosis of ADRD than cisgender (non‐transgender) adults (2.2%) (Guo et al. 2022). Similar findings were reported by researchers examining 2015 Medicare claims data (Dragon et al. 2017). Dragon et al. (2017) discovered that TGN adults had a higher prevalence rate of ADRD when compared to cisgender beneficiaries: 18.2% versus 12.2%. These two studies show that TGN adults have a higher prevalence of ADRD compared to cisgender adults.

Though research is limited, studies have shown that SGM adults are more at risk for SCD than non‐SGM adults (Alzheimer's Association 2023). SCD, or self‐reported experiences of memory loss and/or cognitive impairment that has worsened over the previous 12 months, is a major warning sign and is considered one of the first clinical manifestations of ADRD (Alzheimer's Association 2023; Baiden et al. 2022). Using data from the 2015–2018 Behavioral Risk Factor Surveillance System (BRFSS), Flatt et al. (2021) found that SGM adults were more likely to report SCD than their non‐SGM counterparts. The prevalence of SCD was higher among the TGN adults included in this study (17.3%) when compared to cisgender men (10.7%) and cisgender women (10.4%) (Flatt et al. 2021). In addition, a recent study conducted by Cicero et al. (2023), using 2015–2020 BRFSS data, explored differences in the prevalence of SCD among cisgender and TGN adults from minoritized ethnoracial groups (American Indian or Alaska native, Asian, Black, Hispanic, and other people of color) and White cisgender and TGN adults. TGN adults from minoritized ethnoracial groups had the highest prevalence of SCD (21.6%) followed by White TGN adults (15.0%), while White cisgender adults had the lowest SCD prevalence (9.0%) (Cicero et al. 2023). Lastly, a study done by Lambrou et al. (2022) looked at the association between discrimination faced by TGN adults and increased SCD. Lambrou et al. (2022) found that TGN adults who reported experiencing discrimination in a medical setting were 7.5 times more likely to report memory problems and were 5.3 times more likely to report that their memory worsened over time.

ADRD is also associated with ACEs, which are traumatic and stressful experiences that occur before an individual reaches the age of 18. These experiences can include physical and emotional abuse, neglect, household violence, parental or caregiver addiction, and more. Experiencing one or more ACEs negatively impacts an individual's health, psychosocial well‐being, and educational attainment (Dosanjh, Hinds, and Cubbin 2023). For example, adults with a greater exposure to ACEs have an increased risk of being diagnosed with chronic health conditions, such as coronary heart disease, stroke, asthma, chronic obstructive pulmonary disease, cancer, obesity, and depression when compared to adults who did not experience any ACEs (Merrick et al. 2018). Some of the chronic health conditions that may arise from experiencing ACEs are associated with an increased risk of ADRD (Alzheimer's Association 2023), and ACES are also associated with an increased risk of SCD in cisgender heterosexual adults, but no study has examined this among TGN adults (Baiden et al. 2022; Flatt et al. 2022).

SGM people experience more ACEs compared to their cisgender and/or heterosexual counterparts (Craig et al. 2020; Schnarrs et al. 2019; Tran et al. 2023). However, few studies focus on TGN people or disaggregate study findings relevant to TGN communities from the broader SGM population. To our knowledge, no study has examined the relationship between ACEs and SCD among TGN adults. The purpose of this study is to determine if there is a relationship between ACEs and SCD among TGN adults. We hypothesize that the prevalence of ACEs among TGN communities will be high and that there will be a strong association between ACEs and SCD. Examining how ACEs impact different aspects of health, including brain health, is important to nursing practice as it can provide clinical care strategies and identify interventions to specifically address ways to improve the health and well‐being of TGN adults. Findings from this research can support the delivery of gender‐affirming and trauma‐informed care that is patient‐centered, which respects the patients' needs and desires while protecting their autonomy in decision‐making. By having more comprehensive understanding about the health of the TGN adult population, nurses and other healthcare providers can provide equitable and necessary care in ways that not only affirms the patient's gender but are also grounded in the understanding of how past childhood experiences may affect the overall health of TGN adults.

## 2 Methods

### 2.1 Design

To examine the relationship between ACEs and SCD among TGN adults, we analyzed pooled data from the Centers for Disease Control and Prevention's (CDC) 2019–2021 BRFSS. BRFSS is a cross‐sectional survey conducted annually by each US state and territory via telephone with noninstitutionalized adults aged 18 and older (Centers for Disease Control and Prevention 2019). Data collected by BRFSS are used to help inform public health practices. The BRFSS survey covers a multitude of topics regarding health‐related behaviors and conditions. BRFSS interviews begin with survey items that are asked by every US state and territory, and these items are followed by “Optional Modules” that states opt to include in their BRFSS survey, such as questions about sexual orientation and gender identity (SOGI), memory problems, and ACEs (CDC 2019). The final survey items are state‐added questions.

We included data from TGN BRFSS participants aged 45 years or older who were residing in one of 16 US states that used optional BRFSS modules assessing gender identity, ACEs, and SCD (Florida, Hawaii, Idaho, Iowa, Kansas, Mississippi, New York, Ohio, Oklahoma, Rhode Island, South Carolina, Tennessee, Utah, Virginia, West Virginia, and Wisconsin). The gender identity survey item from the SOGI optional module was used to identify TGN participants (CDC 2019). BRFSS participants who answered “yes” to “Do you consider yourself to be transgender?” were categorized as TGN in our study. This secondary analysis of publicly available, de‐identified data did not require a review by an institutional review board.

### 2.2 Measures

Demographic characteristics. Demographic measures included self‐reported age in years, race/ethnicity, highest grade or year of school completed, and current employment status. For educational attainment, we categorized individuals who indicated that they never went to school or only attended kindergarten, elementary, or some high school as “less than a high school diploma;” those with a grade 12 education or obtained a GED as “high school graduate;” those who attended 1–3 years of college as “some college or technical school” and those who attended college for 4 years or more as a “college graduate.” Income was not used as a covariate because 17% of the sample had missing income data.

#### 2.2.1 Adverse Childhood Experiences

In 2009, BRFSS began offering an optional module to collect data about traumatic childhood events that may have been experienced before the age of 18 (Centers for Disease Control and Prevention 2023). These data were not made publicly available until the 2019 BRFSS; as such, this study included ACE data from the 2019–2021 BRFSS. The ACE optional module consisted of 11 survey items that assessed a range of traumatic childhood events with response options varying from “yes, no, don't know or not sure” to “never, once, more than once, don't know or not sure.” Each ACE item was dichotomized to indicate exposure to the ACE item as indicated by a “yes” or “once, more than once” response. For our analyses and consistent with prior research (Felitti et al. 1998; Suarez et al. 2021), we created an ACE sum score, a continuous measure that indicated the total number of ACEs a participant experienced, and two categorical variables: one represented the number of ACEs experienced (0, 1, 2, 3, or 4 or more ACEs) and the other grouped ACEs into two categories—childhood abuse (verbal, physical, and sexual abuse) and household dysfunction (living with an adult with mental illness, history of alcohol or substance abuse, or incarceration; witness physical abuse; parents separated or divorced).

#### 2.2.2 Subjective Cognitive Decline

The “Cognitive Decline” optional module was asked to BRFSS participants aged 45+ (Centers for Disease Control and Prevention 2020). Participants endorsed SCD by affirming that during the past 12 months, they experienced confusion or memory loss that was happening more often or getting worse.

### 2.3 Statistical Analysis

Descriptive statistics were used to detail sample characteristics including means, medians, and standard deviations for continuous variables and frequencies and percentages for categorical variables. Pearson's chi‐squared tests assessed the association between ACEs (individual, categorical, and sum score) and SCD. If expected cell counts were less than five, Fisher's exact test was used to assess this association. Crude and adjusted odds ratios (OR) along with 95% confidence intervals (CI) were obtained from logistic regression models that investigated the relationship between each ACE and SCD. Analyses were repeated with the ACE categorical variable, and we used the ACE sum score, due to sample size restraints, to examine a dose–response relationship. Separate logistic regression models were fit for each ACE variable examined. Due to concerns about the application of BRFSS sampling weights in TGN analyses (Cicero et al. 2020a, 2020b; Lett and Everhart 2022), sampling weights were not included in our models. Models were adjusted for BRFSS year, age, race, education, and employment. Statistical tests were performed using SAS 9.4 with the level of significance set at 0.05 for each test, which was not adjusted for multiple tests due to the exploratory nature of this study.

## 3 Results

The study included 206 TGN adults aged 45 and older. The sample was predominantly White, non‐Hispanic (71%), had at least a high school education (86%), and was retired (43%). In addition to sample demographic characteristics, Table 1 reports the prevalence of SCD and ACEs. Approximately 18% (*n* = 38) of TGN adults endorsed SCD. TGN adults reported an average of 1.8 ACEs, and 41% (*n* = 85) reported never experiencing an ACE while nearly 20% reported experiencing one ACE (*n* = 41) or four or more ACEs (*n* = 37, 18%). Nearly half of the sample experienced childhood abuse (*n* = 94) and household dysfunction (*n* = 92). Within the childhood abuse category, “verbal abuse” (*n* = 64, 31%) and “physical abuse by a parent or an adult in the home” (*n* = 56, 28%) were the most prevalent with “forced to have sex with a parent or an adult in the home” (*n* = 13, 6%) being the least. For household dysfunction, “living with someone who was a problem drinker or alcoholic” (*n* = 43, 21%) was the most prevalent, and “living with someone who was incarcerated” (*n* = 14, 7%) was the least.

**TABLE 1:** Demographic characteristics and prevalence of SCD and ACEs among transgender TGN adults ages 45+ (*N* = 206).

Variable	*N* (%)
Demographic characteristics	
Age (years), mean (SD, median, range)	64.54 (10.68, 64.50, 45–80)
Race/ethnicity	
American Indian or Alaskan Native only, non‐Hispanic	6 (2.91)
Asian, Native Hawaiian or other Pacific Islander only, non‐Hispanic	11 (5.34)
Black only, non‐Hispanic	21 (10.19)
Hispanic	12 (5.83)
Another race or multiracial, non‐Hispanic	10 (4.85)
White only, non‐Hispanic	146 (70.87)
Educational attainment	
Less than a high school diploma	28 (13.66)
High school diploma or GED	72 (35.12)
Some college or technical school	52 (25.37)
College graduate	53 (25.85)
Employment status	
Employed/self‐employed	67 (32.84)
Unemployed	9 (4.41)
Homemaker	3 (1.47)
Student	3 (1.47)
Retired	88 (43.14)
Unable to work	34 (16.67)
Annual household income	
< $20k	44 (25.73)
$20k to < $50k	72 (42.11)
> $50k	55 (32.16)
Subjective cognitive decline	*N* = 204
Yes	38 (18.45)
Adverse childhood experiences	
Number of ACEs^a^ (Mean ([SD, median, range)]	1.79 (2.33, 1.00, 0–9)
0	85 (41.26)
1	41 (19.90)
2	22 (10.68)
3	21 (10.19)
4 or more	37 (17.96)
Any ACE	120 (59.70)
Childhood abuse	94 (46.53)
Verbally abused by parents/adults in home	64 (31.37)
Physically abused by parents/adults in home	56 (27.72)
Sexually abused	26 (12.81)
Forced to touch someone sexually	20 (9.71)
Forced to have sex	13 (6.34)
Household dysfunction	92 (46.00)
Lived with someone with mental illness	36 (17.91)
Lived with someone who was a problem drinker/alcoholic	43 (20.98)
Lived with someone who abused drugs	19 (9.31)
Lived with someone who was incarcerated	14 (6.83)
Parents were separated or divorced	41 (20.50)
Parents/adults in home physically abused each other	33 (16.58)

Abbreviations: ACEs, adverse childhood experiences; BRFSS, Behavioral Risk Factor Surveillance System; GED, general educational development; SCD, subjective cognitive decline; SD, standard deviation.

^a^ Data represent the 11 ACE survey items asked in the 2019–2021 BRFSS.

The prevalence of ACEs among TGN adults endorsing SCD and their association with ACEs is detailed in Table 2. Among those endorsing SCD (*n* = 38), 18% (*n* = 7) reported no ACE experiences, whereas 34% (*n* = 13) reported four or more ACEs. Approximately 73% of TGN adults with SCD also experienced childhood abuse (*n* = 26) or household dysfunction (*n* = 27). For childhood abuse, TGN adults with SCD were more likely to report verbal abuse (*n* = 19, 51%) and least likely to report forced sex (*n* = 5, 13%). Among household dysfunction ACEs, TGN adults with SCD were more likely to report living with someone who was a problem drinker or alcoholic (*n* = 14, 38%) and least likely to report living with someone who was incarcerated (*n* = 6, 16%). Most ACEs were associated with an increased risk of SCD. The odds of SCD increased by 32% and 38% as the number of ACEs experienced increased in our unadjusted (95% CI: 1.15–1.51, *p* < 0.0001) and adjusted models (95% CI: 1.18–1.62, *p* < 0.0001), respectively. In our models adjusted for BRFSS year, age, race, education, and employment, there were increased odds of SCD that ranged from 2.8 to 4.3 for all ACEs except for two, “forced to touch someone sexually” and “forced sex.” The odds of SCD were higher if a TGN adult experienced childhood abuse (aOR = 4.3, 95% CI: 1.88–10.02, *p* < 0.01) or household dysfunction (aOR = 4.7, 95% CI: 2.00–11.07, *p* < 0.01).

**TABLE 2:** Prevalence and association of ACEs and SCD among transgender adults living with SCD.

Adverse childhood experience	Living with SCD (*N* = 38), *n* (%)	Unadjusted odds ratio (95% confidence interval)	Adjusted odds ratio^a^ (95% confidence interval)
Number of ACEs			
Sum score	—	1.32 (1.15, 1.51)***	1.38 (1.18, 1.62)***
0	7 (18.42)**	—	—
1	7 (18.42)	—	—
2	5 (13.16)^b^	—	—
3	6 (15.79)^b^	—	—
4 or more	13 (34.21)**	—	—
Any ACE	31 (83.78)***		
Childhood abuse	26 (72.22)**	3.75 (1.70, 8.27)**	4.34 (1.88, 10.02)**
Verbally abused by parents/adults in home	19 (51.35)**	2.86 (1.38, 5.94)**	3.35 (1.52, 7.41)**
Physically abused by parents/adults in home	17 (48.57)**	3.10 (1.46, 6.59)**	3.16 (1.44, 6.96)**
Sexually abused	9 (24.32)*, ^b^	2.82 (1.14, 6.95)^b^	2.88 (1.12, 7.39)^b^
Forced to touch someone sexually	7 (18.42)	2.69 (0.99, 7.29)	2.73 (0.93, 8.00)
Forced to have sex	5 (13.16)	3.01 (0.93, 9.79)	2.73 (0.72, 10.31)
Household dysfunction	27 (72.97)**	4.07 (1.85, 8.97)**	4.71 (2.00, 11.07)**
Lived with someone with mental illness	13 (36.11)**	3.49 (1.55, 7.85)**	4.29 (1.76, 10.49)**
Lived with someone who was a problem drinker/alcoholic	14 (37.84)**	2.92 (1.34, 6.34)**	3.24 (1.40, 7.52)**
Lived with someone who abused drugs	7 (18.92)^b^	3.01 (1.10, 8.28)^b^	3.20 (1.07, 9.55)^b^
Lived with someone who was incarcerated	6 (16.22)*, ^b^	3.87 (1.26, 11.94)^b^	3.80 (1.14, 12.65)^b^
Parents were separated or divorced	13 (36.11)^b^	2.75 (1.24, 6.06)^b^	2.75 (1.17, 6.45)^b^
Parents/adults in home physically abused each other	12 (33.33)**	3.38 (1.47, 7.76)**	3.22 (1.32, 7.90)^b^

^a^ Adjusted for BRFSS year, age, race, education, and employment.

^b^ Fisher's exact test was performed.

* *p* < 0.05.

** *p* < 0.01.

*** *p* < 0.001.

## 4 Discussion

To our knowledge, this is the first study to examine the relationship between ACEs and SCD among TGN adults, an underrepresented population in ADRD clinical research. Our findings suggest that most TGN adults in our sample (59.7%) experienced one or more ACEs before the age of 18, which are experiences representing both household dysfunction and childhood abuse. Nine of the 11 ACEs examined were associated with SCD and the odds of SCD were higher among TGN adults who experienced ACEs compared to those who did not. This increase in odds ranged from 2.8 to 4.3 in our adjusted models. In our models, as the number of ACEs experienced increased, the odds of SCD also increased. These findings support our hypotheses that among TGN adults, ACEs are highly prevalent, and they increase the risk of SCD. Although this was the first examination of the association between ACEs and SCD among TGN adults, our findings align with previous studies that found a dose–response relationship between ACEs and SCD among cisgender and sexual minority adults (Baiden et al. 2022; Brown et al. 2023, 2022; Terry et al. 2023).

Our findings demonstrated an increase in the likelihood of SCD as the number of ACES experienced increased. This finding is consistent with prior research in which there was an associated increase in the likelihood of being diagnosed with various health conditions as the number of ACES experienced increased. For example, research examining the health of TGN adults has shown a relationship between ACEs and depression (Suarez et al. 2021; Tran et al. 2023), poor mental health (King et al. 2024; Tran et al. 2023), poor physical health, suicidality, and post‐traumatic stress disorder (Suarez et al. 2021). In addition to these health conditions, a study that examined ACEs among transmasculine adults found that as the number of ACEs increased, transmasculine individuals were more likely to report adverse health behaviors including alcohol and substance abuse, smoking, and obesity, which are ADRD risk factors (Suarez et al. 2021). Findings from previous research and our current study, all of which are cross‐sectional, illuminate an association between ACEs and the diagnosis of ADRD risk factors later in life among TGN adults. Further research is needed to examine causality between ACEs and ADRD and ADRD risk factors.

The TGN adults in our sample reported a high burden of ACEs. This finding is similar to previous studies among TGN individuals (King et al. 2024; Schnarrs et al. 2019; Suarez et al. 2021; Tran et al. 2023), yet when compared to the general population, our findings point to inequities among the TGN population. In our sample, nearly 60% of TGN adults reported experiencing at least one ACE, compared to 52% of adults enrolled in the Kaiser Health Plan in San Diego (Felitti et al. 1998) and 24% of adults from a nationally representative US sample (Merrick et al. 2018). Moreover, the proportion of TGN adults in our sample who experienced four or more ACEs was three times greater than what was found by Felitti et al. (1998). Although our study focused on TGN adults aged 45+, previous research among TGN and cisgender adults of all ages reported similar TGN inequities. For example, TGN individuals were more likely to report higher ACE scores (Craig et al. 2020; Tran et al. 2023) and were more likely to report emotional abuse, emotional neglect, and physical abuse (Schnarrs et al. 2019) compared to cisgender people. Further research is needed to explore ACEs and their association with SCD in specific TGN communities such as transgender women, transgender men, and nonbinary people. Moreover, research that applies an intersectional lens is critically important because ACEs and SCD disproportionally impact TGN people of color compared to White TGN and cisgender communities as well as cisgender communities of color (Cicero et al. 2023; King et al. 2024). Additionally, future research is needed to examine the relationship between ACEs and SCD as well as other health outcomes among TGN adults living outside of the United States.

### 4.1 Nursing Implications

Examining the association between ACEs and SCD among TGN adults is important to nursing practice as interventions and care strategies can be identified to improve the overall health of TGN adults. Given our research findings and the health implications associated with ACEs, we encourage universal screening for ACEs. In addition, screening for SCD and other memory issues should also be implemented if the patient has previously endorsed having experienced ACEs. By being able to detect an increased risk for ADRD, preventive interventions and treatments may be employed. Nurses and other healthcare providers should work together to provide care that is equitable and gender‐affirming with the knowledge of how childhood experiences have a lasting and significant impact on the health of TGN adults. Nursing care should also be delivered using a trauma‐informed approach to prevent re‐traumatization (Cicero et al. 2024). TGN adults often experience stigma from discrimination at the structural, interpersonal, and individual levels, which results in discrimination‐related trauma and is associated with health and social inequities (Cicero et al. 2024). This emphasizes the importance of educating nurses in the delivery of trauma‐informed, patient‐centered, and gender‐affirming healthcare. During healthcare encounters, all patients, especially TGN people, should feel empowered and given autonomy in decisions related to their care.

### 4.2 Limitations

There are several limitations to this study. Although we pooled data from multiple BRFSS years, the study was cross‐sectional, and we were unable to examine causation or determine how the BRFSS participants identified their gender during childhood. Results from our study are only representative of the 16 US states that administered the optional SOGI, ACEs, and SCD modules from 2019 to 2021, and may not be generalized to all TGN adults in the United States. Our study relied on data from these optional BRFSS modules, which resulted in a small analytic sample of TGD adults (*N* = 206) meeting our study inclusion criteria. Responses to BRFSS modules, including the three optional modules, are self‐reported and rely solely on participants' memory and recall, which may introduce bias and lead to underreporting. Participants may have also felt uncomfortable answering questions regarding their gender identity and past childhood experiences, which can be very personal, and may have resulted in underreporting of the number of TGN adults as well as their ACE experiences. The BRFSS optional ACE module used a general approach to understanding childhood experiences, and the survey items were not tailored to TGN‐specific experiences of abuse or mistreatment associated with an individual's gender identity or gender expression. Whether their experience of ACEs was due to identifying as TGN or if the ACEs were unrelated to their gender identity cannot be determined. This represents an area for future research. Additionally, our study only examined ACEs and its relationship with SCD, a risk factor for ADRD. Our study did not account for other factors, such as stress, sleep habits, diet, exercise, environment, substance use, that may also contribute to SCD. Despite the limitations described, our study contributes to current knowledge about the relationship between ACEs and SCD and its impact on the brain health of TGN adults.

## 5 Conclusion

ACEs increase the risk of SCD, an early clinical manifestation of ADRD. Screening for ACEs is important as they are associated with negative health outcomes. With this knowledge, nurses and other healthcare providers can create and implement a care plan that is patient centered. Nurses and other healthcare providers should work together to provide care that is equitable and gender‐affirming with the knowledge of how childhood experiences have a lasting and significant impact on the health of TGN adults.

## 6 Clinical Resources

- Alzheimer‘s Association. Alzheimer‘s and dementia. https://www.alz.org/alzheimer_s_dementia
- Centers for Disease Control and Prevention. Maintaining your brain health. https://www.cdc.gov/aging/olderadultsandhealthyaging/maintainng‐your‐brain‐health.html
- Centers for Disease Control and Prevention. Preventing adverse childhood experiences (ACEs): Leveraging the best available evidence. https://stacks.cdc.gov/view/cdc/82316
- National League for Nursing. Advancing Care Excellence for the LGBTQ 1 People (ACE+). https://www.nln.org/education/teaching‐resources/professional‐development‐programsteachingresourcesace‐all/aceplus
- National LGBT Health Education Center at The Fenway Institute. https://fenwayhealth.org/the‐fenwayinstitute/education/the‐national‐lgbtia‐healtheducation‐center/
- Sexual Orientation and Gender Identity Nursing. https://soginursing.ca
- World Professional Association for Transgender Health. www.wpath.org

## Conflicts of Interest

Dr. Cicero was supported by grants from the Alzheimer's Association (23AARGD‐NTF‐1028973), the National Institute on Alcohol Abuse and Alcoholism (R01AA030275), and the National Institute on Aging (K23AG084851). The statements in this article are solely the responsibility of the author and do not necessarily represent the views of the Alzheimer's Association or the National Institutes of Health.

## Sigma Theta Tau International Chapter

Alpha Epsilon Chapter.

## Data Availability

Data used in these analyses are publicly available online from the Centers for Disease Control and Prevention's Behavioral Risk Factor Surveillance System.
